# Telomere Length in Valve Tissue Is Shorter in Individuals With Aortic Stenosis and in Calcified Valve Areas

**DOI:** 10.3389/fcell.2021.618335

**Published:** 2021-03-11

**Authors:** Ilona Saraieva, Athanase Benetos, Carlos Labat, Anders Franco-Cereceda, Magnus Bäck, Simon Toupance

**Affiliations:** ^1^INSERM, DCAC, Université de Lorraine, Nancy, France; ^2^CHRU-Nancy, Pôle “Maladies du Vieillissement, Gérontologie et Soins Palliatifs”, Université de Lorraine, Nancy, France; ^3^Karolinska University Hospital, Theme Heart and Vessels, Stockholm, Sweden; ^4^Department of Molecular Medicine and Surgery, Karolinska Institutet, Stockholm, Sweden; ^5^Department of Medicine, Karolinska Institutet, Solna, Stockholm, Sweden

**Keywords:** telomere length, aortic valve, aortic stenosis, calcification, aging

## Abstract

**Background:**

Short telomere length (TL) is associated with age-related diseases, in particular cardiovascular diseases. However, whether the onset and course of aortic stenosis (AS) is linked to TL in aortic valves remains unknown.

**Objectives:**

To assess telomere dynamics (TL and telomerase activity) in aortic valves and the possible implication of TL in onset and course of AS.

**Methods:**

DNA was extracted from aortic valves obtained from 55 patients (78.2% men; age, 37–79 years), who had undergone replacement surgery due to AS (AS group, *n* = 32), aortic valve regurgitation and aortic dilation (Non-AS group, *n* = 23). TL was measured by telomere restriction fragment analysis (TRF) in calcified and non-calcified aortic valve areas. Telomerase activity was evaluated using telomerase repeat amplification protocol (TRAP) in protein extracts from non-calcified and calcified areas of valves obtained from 4 additional patients (50% men; age, 27–70 years).

**Results:**

TL was shorter in calcified aortic valve areas in comparison to non-calcified areas (*n* = 31, 8.58 ± 0.73 kb vs. 8.12 ± 0.75 kb, *p* < 0.0001), whereas telomerase activity was not detected in any of those areas. Moreover, patients from AS group displayed shorter telomeres in non-calcified areas than those from the Non-AS group (8.40 ± 0.64 kb vs. 8.85 ± 0.65, *p* = 0.01).

**Conclusions:**

Short telomeres in aortic valves may participate in the development of AS, while concurrently the calcification process seems to promote further local decrease of TL in calcified areas of valves.

## Introduction

Aortic stenosis (AS), the most common valvulopathy among the aging population in developed countries, affects both tricuspid and bicuspid valves (Roberts and Ko, 2005; Huntley et al., 2018). Regardless of congenital abnormalities, the prevalence of AS increases with age (Roberts and Ko, 2005) and while estimated at 0.4% in the general adult population, it rises to 2.85% in subjects over 75 years (Nkomo et al., 2006). Degenerative AS, as a result of calcific aortic valve disease (CAVD) (Yutzey et al., 2014; Lerman et al., 2015), is characterized by progressive cusp calcification (Yutzey et al., 2014; Lerman et al., 2015). Calcific deposition in the leaflets is an active and highly regulated process, which involves mechanisms similar to those of atherosclerotic cardiovascular disease (ASCVD) (Mathieu and Boulanger, 2014; Pawade et al., 2015). Indeed, the pathogenesis of both diseases include lipid infiltration, chronic inflammation, and calcification (Mathieu and Boulanger, 2014; Pawade et al., 2015). In addition, CAVD shares many risk factors with ASCVD, such as hypertension (Stewart et al., 1997), diabetes (Larsson et al., 2018), obesity (Larsson et al., 2020), smoking (Larsson et al., 2017) and chronic kidney disease (Vavilis et al., 2019).

An increasing number of epidemiological studies focusing on telomere length (TL) have found that ASCVD and its risk factors are associated with short leukocyte TL (LTL) (Valdes et al., 2005; Haycock et al., 2014; D’Mello et al., 2015; Verhulst et al., 2016; Toupance et al., 2017; Benetos et al., 2018). Moreover, it was proposed that short LTL may be established early in life and precede the development of ASCVD (Benetos et al., 2019). Taken together these data suggest a role for TL in atherogenesis and, in parallel, potentially in CAVD and AS development. However, very little is known about the implication of TL in aortic valve calcification. Kurz et al. (2006) reported that short LTL was associated with AS in individuals older than 70 years, but no one, to the best of our knowledge, has ever studied the relation between CAVD and TL measured directly in aortic valves. Thus, the aim of the present study was to assess telomere dynamics (TL and telomerase activity) in aortic valves and its possible implication in AS and CAVD.

## Materials and Methods

### Sample Collection

Aortic valves were obtained from patients (*n* = 83) undergoing aortic valve replacement for AS, aortic valve regurgitation, and aortic dilation at the Karolinska University Hospital (Stockholm, Sweden). The study was approved by the local ethical committee (2012/1633) and informed consent was obtained from all patients.

After surgical removal, valve leaflets were immediately immersed in a preservation liquid and stored at 4°C until shipped to the laboratory. For TL measurements, valves were collected in RNA Later solution (Qiagen, Hilden, Germany) and for telomerase activity determinations in phenol red-free DMEM supplemented with 10% fetal bovine serum. In the laboratory, samples were stored at −80°C until analysis.

Among the 83 valves, 79 were used for TL measurements and 4 for telomerase activity determination. In this study we used valves for which we obtained DNA at least for non-calcified valve areas. Thus, after DNA extraction, 24 valves were excluded due to insufficient amount or poor DNA quality (Supplementary Figure 1). In the subsequent analyses, valves were divided into two groups according to the diagnosis:

- AS group: aortic valves obtained from patients with a diagnosis of AS as the indication for surgery (*n* = 32).
- Non-AS group: valves obtained from patients without a diagnosis of AS, undergoing surgery for other indications (*n* = 23). This group included diagnoses of aortic regurgitation, and aortic dilation and was further dived into aortic sclerosis if valves displayed macroscopic signs of calcification (*n* = 7) and non-sclerosis for macroscopically normal valves (*n* = 16).

### Macroscopical Dissection of Aortic Valves

Each valve was evaluated macroscopically for the presence of normal, thickened and calcified areas and then dissected based on this assessment, as described previously (Nagy et al., 2011). In addition, a subset of non-sclerotic valves was dissected by separating the tip and the base of each aortic valve cusp.

These dissections allowed the comparison of TL in aortic valve tissue between different areas within the same valve (Supplementary Figure 2). Comparisons were performed in three situations. First, we compared TL between normal and thickened tissues derived from *n* = 10 valves. This analysis showed no significant difference between these two types of aortic valve tissues (Supplementary Figure 3A), which allowed us to regroup normal and thickened under the common term non-calcified tissue. Subsequently, we performed TL comparisons between non-calcified and calcified tissues within the same valve (*n* = 31). Third, we compared TL between the tip and base (*n* = 6) in non-sclerotic valves. The aim of this comparison was to study the influence of the anatomical areas on TL in the absence of macroscopic calcifications.

For some valves, DNA for TL measurements was obtained only from one tissue area; these valves were not included in the comparisons but used in other analyses (Supplementary Figure 2).

To assess TL independently of the presence of local calcifications, we used non-calcified areas from valves to compare between AS and Non-AS groups. The same valve areas were used also to assess the relations of TL with age, sex and bicuspidy.

### Telomere Length Measurements

DNA was extracted from valves with the phenol/chloroform/isoamyl alcohol method after grinding up tissues in liquid nitrogen. Prior to the analysis, DNA quantity was assessed by spectrophotometry and DNA samples passed an integrity testing using a 1% (wt/vol) agarose gel. TL measurements were performed by Southern blot analysis of the terminal restriction fragments as described previously (Kimura et al., 2010). Briefly, DNA samples were digested (37°C) overnight with restriction enzymes *Hin*fI (10U)/*Rsa*I (10U) (Roche Diagnostics GmbH, Mannheim, Germany). Digested DNA samples and DNA molecular weight ladders were then resolved in 0.5% (wt/vol) agarose gels for 24 h at 40 V. After electrophoresis, the DNA was depurinated, denatured, neutralized and then blotted onto positively charged nylon membranes (Roche Diagnostics GmbH) by vacuum transfer (Bio-Rad, Hercules, CA, United States). Hybridization was performed at 42°C overnight with a digoxigenin-labeled telomeric probe after 1 h of prehybridization. Membranes were washed, blocked for proteins and incubated with an alkaline phosphatase conjugated anti–digoxigenin-antibody. Probe-target hybrids were detected using alkaline phosphatase conjugate cleavage of chemiluminescent CDP-Star substrate solution (Roche Diagnostics GmbH). A CCD camera (Las 4000, Fujifilm Life Sciences, Cambridge, MA, United States) was used for visualization of the chemiluminescence signal.

Different samples from the same individual were always run in adjacent lanes on the same membrane; for samples with sufficient amount of DNA (*n* = 70), measurements were performed in duplicate on separate membranes. The inter-assay coefficient of variation for the duplicate measurements was 2.02%.

### Telomerase Activity Determination

Telomerase activity was evaluated by the modified telomerase repeat amplification protocol (TRAP) (Krupp et al., 1997). Measurements were performed for non-calcified and calcified areas of 4 valves obtained from 2 men and 2 women in the age range 27–70 years. Briefly, tissue specimens were snap-frozen and ground up in liquid nitrogen followed by lysis in CHAPS buffer for 20 min on ice. After incubation, lysates were centrifuged at 13,000×*g* for 20 min at 4°C. The supernatant was collected into new tubes and protein concentration was measured using the Quick Start^TM^ Bradford Protein Assay (Bio-Rad). A protein extract from HeLa cells, which contain an active telomerase, was used as a positive control. For each sample, 50 μl of reaction mix was performed. Each reaction contained TRAP reaction buffer (final concentration: 20 mM Tris-HCl (pH 8.3), 1.5 mM MgCl_2_, 63 mM KCl, 1 mM EGTA, 0.005% Tween 20), 50 μM dNTPs, 22.5 pmol primer TS (5′-AATCCGTCGAGCAGAGTT), 22.5 pmol primer CXext (5′-GTGCCCTTACCCTTACCCTTACCCTAA), 2 units of fast-start Taq-polymerase (Roche Diagnostics GmbH) and 1 μg of protein extract. The TRAP reaction consisted of initial incubation at 37°C for 30 min for TS primer extension by telomerase, followed by deactivation of the telomerase and activation of Taq-polymerase for 2 min at 94°C and 33 amplification cycles (30 s 94°C, 30 s 52°C, 30 s 72°C) with a final extension step of 10 min at 72°C. PCR products were resolved on a 12% non-denaturing polyacrylamide gel (19:1) and stained with 1 × SYBR Green I (Thermo Fisher Scientific, Waltham, MA, United States). DNA bands were visualized with UV on a GelDoc imager (Bio-Rad).

In order to rule out false positive and negative results, two additional control reactions were assayed alongside each sample. First control, applied to detect false positive results due to primer-dimer formation during PCR, contained heat inactivated protein at 95°C for 10 min before subjecting it to the TRAP reaction. Second control, designed to avoid false negative results caused by PCR inhibitors in tissue extracts, consisted of 60 ng of protein extract from HeLa cells added with each sample of interest before subjecting it to the TRAP reaction.

### Statistical Analysis

Discrete variables are presented as percentages and continuous variables as mean ± SD. The characteristics of the patients were compared using the Mann–Whitney U and χ^2^-tests, as appropriate. Comparisons between patient groups were performed using a two-sample *t*-test. Adjustments for age and sex were made by using a general linear model. Differences in TL between tissue areas were evaluated with paired *t*-test. Bivariate relations between continuous variables were determined using Pearson correlation coefficients. *P* < 0.05 was considered as statistically significant. The software packages used to analyze the data were NCSS 9 (NCSS, Kaysville, UT, United States) and GraphPad Prism 8.4.0 (GraphPad, San Diego, CA, United States).

## Results

### Patient’s Characteristics

Overall, TL was measured in aortic valves from 55 patients (Supplementary Figure 1). Among them, 32 patients were in the AS group and 23 in the Non-AS group. Characteristics of all individuals are presented in Table 1. No differences were observed in age and sex between the two groups. All patients but one in the AS group presented macroscopical calcifications. In the Non-AS group, 7 patients displayed aortic sclerosis.

**TABLE 1:** Characteristics of the patients.

	All *n* = 55	AS group *n* = 32	Non-AS group *n* = 23	*P*
Age, years	64.7 ± 9.5	63.3 ± 10.0	66.7 ± 8.6	0.21
Men (%)	78.2	75.0	82.6	0.50
Tricuspid valve (%)	43.6	28.1	65.2	<0.01
Macroscopical calcification (%)	69.0	96.9	30.4	<0.0001

**Values are expressed as either mean ± SD or %. P-values were calculated using χ^2^-test for discrete variables and Mann–Whitney test for age (non-normal distribution). P < 0.05 was considered as significant.**

### Telomere Length Dynamics Within Different Areas of Aortic Valve

**FIGURE 1:**
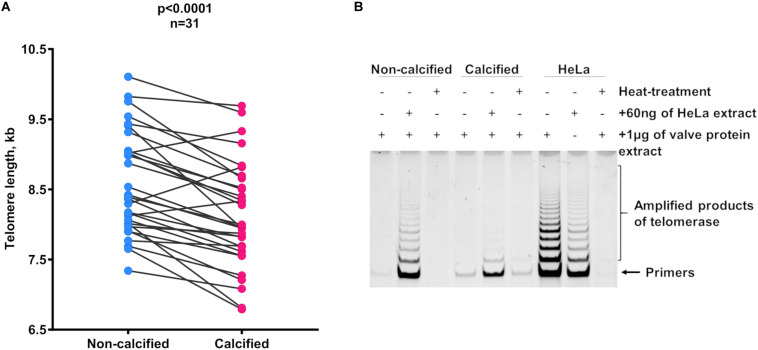
Telomere length dynamics in different areas of the AS and sclerotic valves. **(A)** Telomere length in non-calcified and calcified areas. Matched circles stand for individual patients. Statistical analysis was performed using paired *t*-test. **(B)** Representative picture of telomerase activity evaluation by TRAP in non-calcified and calcified areas of the valve tissue (*n* = 4 for each type of tissue). Lines correspond to telomerase activity in 1 μg of protein extract. Samples, which contain HeLa cell extract mixed with tissue extract, represent controls for false-negative results and heat-treated samples serve as controls for false-positive results. Extracts from HeLa cells were used as the positive control. kb, kilobase.

Calcified areas of aortic valves had significantly shorter telomeres than non-calcified areas. The gap between the means of the two areas was 0.46 kb (non-calcified: 8.58 ± 0.73 kb; calcified: 8.12 ± 0.75 kb; *p* < 0.0001; Figure 1A). Notably, no telomerase activity was detected either in non-calcified or in calcified areas (Figure 1B). This lack of telomerase activity was observed in all samples, either bicuspid or tricuspid valves, sclerotic, or stenotic valves originating from patients of different ages (27–70 years-old, Supplementary Figure 4). In addition, the comparison between the base, a more calcification-prone area (Otto et al., 1994), and the tip of non-sclerotic aortic valves did not reveal any difference in TL between these two anatomical areas (Supplementary Figure 3B).

### Comparison of TL Between AS and Non-AS Patients

**FIGURE 2:**
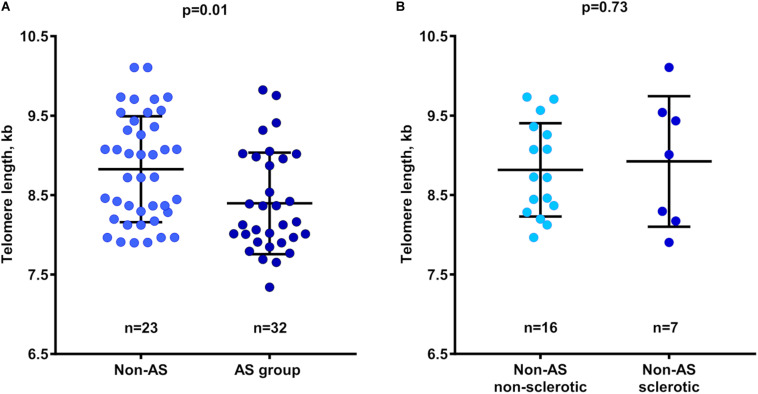
Relation between telomere length in non-calcified valve tissues and patient groups. **(A)** Telomere length in Non-AS and AS subjects. **(B)** Telomere length in Non-AS non-sclerotic and Non-AS sclerotic subjects. Data are presented as mean ± *SD* and differences between groups was assessed using two-sample *t*-test. AS, aortic stenosis; kb, kilobase.

TLs in non-calcified aortic valve areas were significantly shorter in the AS compared with the Non-AS group (8.40 ± 0.64 kb vs. 8.85 ± 0.65 kb; *p* = 0.01; Figure 2A). Additional adjustment of TL values for age and sex made this association stronger (*p* < 0.01). Of note, comparison of TL between Non-AS sclerotic and Non-AS non-sclerotic groups revealed no differences (Figure 2B).

### Relation of TL in Non-calcified Areas of Aortic Valves With Age, Sex, and Bicuspidy

TL in non-calcifed areas of aortic valves was not influenced by sex or the presence of bicuspid valve abnormality (Supplementary Figure 5). There was a trend toward shorter TL in older subjects (*p* = 0.13; Supplementary Figure 6).

## Discussion

Two main findings arise from this study. First, TL was shorter in calcified than in non-calcified areas of aortic valves from patients with CAVD. Second, valves derived from patients suffering from AS displayed shorter telomeres also in non-calcified areas as compared to the corresponding valve areas from individuals without AS. Taken together, these data suggest a role of TL in the development of clinically significant AS, as well as a potential local change in TL during the calcification process.

The present study raises a first notion that connects locally accelerated valvular aging with calcification and AS. Indeed, our results revealed that aortic valve areas with macroscopic calcifications displayed shorter telomeres compared with areas without calcifications within the same valve. These findings provide a first suggestion for site-specific telomere dynamics in the aortic valve, which may appear either as a consequence of calcification, or as part of a local valvular aging process that culminates in valve calcification and AS.

To decipher if the biomechanical forces acting on aortic valves (Bäck et al., 2013), which potentially could directly affect TL at different anatomical locations within the valve, the tip and the base were separated from valves macroscopically free of calcification. Given that calcification processes start preferentially at the base of the valve and expands toward the tip (Otto et al., 1994), the lack of significant TL differences between these areas (Supplementary Figure 3B) suggests that differences in valvular TL dynamics are minor in the absence of calcification. Likewise, the lack of detectable telomerase activity in aortic valves points to other underlying mechanisms for the observed local decreased TL with valve calcification in the present study.

Oxidative stress and chronic inflammation can be possible common denominators for TL shortening and early valve changes, since they may cause local telomere attrition and valve calcification (Miller et al., 2008; Branchetti et al., 2013; De Meyer et al., 2018; Mercier et al., 2020). In fact, previous studies in atherosclerotic lesions, which exhibit similar oxidative stress and inflammation as stenotic aortic valves, reported the presence of shorter telomeres compared with healthy vessels (Okuda et al., 2000; Matthews et al., 2006; Nzietchueng et al., 2011). Moreover, smooth muscle cells and endothelial cells of atherosclerotic arteries have been shown to display features of telomere-based senescence (Minamino et al., 2002; Matthews et al., 2006). However, another mechanism can be proposed to explain the decrease in TL observed in calcified tissue. In the present study, TL was measured in bulk tissue areas. Thereby, short telomeres measured in calcified areas can partially reflect increased presence of immune cells, which abundantly infiltrate lesions (Otto et al., 1994; Coté et al., 2013) and have relatively shorter TL than other somatic tissues (Friedrich et al., 2000; Daniali et al., 2013; Benetos et al., 2018).

It has been previously reported that AS patients compared with contemporary controls have shorter telomeres measured in leukocytes (Kurz et al., 2006). Here we report that non-calcified areas in valves derived from patients with AS exhibited shorter telomeres compared with the corresponding tissues in valves derived from patients without clinically significant AS, even in the presence of sclerotic valvular changes. Our findings provide a first suggestion that short TL may precede aortic valve calcification and render the valve more prone to progress to clinically significant AS. This assumption is consistent with a recent study in mice, which suggested TL-dependent gene regulation in aortic valve calcification (Theodoris et al., 2017). Further support comes from results observed in ASCVD patients. Clinical studies reported that individuals with shorter TL were at higher risk of developing ASCVD (Chen et al., 2014) and progressing to advanced stages of the disease over periods of 5 years (Willeit et al., 2010) and 10 years (Toupance et al., 2017). Likewise, shorter TL was associated with early onset ASCVD (Toupance et al., 2017). Moreover, Mendelian randomization studies, using TL-associated single nucleotide polymorphisms, showed that alleles associated with shorter TL are overrepresented in individuals with clinical manifestations of ASCVD (Codd et al., 2013; Scheller Madrid et al., 2016; Haycock et al., 2017), inferring a causal role of short TL in ASCVD development.

As far as we are aware, this is the first clinical study investigating telomere dynamics in human aortic valve tissue. We should however acknowledge that the observational design of the study cannot ascertain causality between TL and either valve calcification or AS. It should be noted also that we detected a low level of inhibition of the TRAP reaction by protein extracts from valve tissues, which might have affected the sensitivity of telomerase detection. In addition, for some sub-group analyses, the modest sample size limited the power to detect differences.

## Conclusion

In conclusion, this study demonstrates that short telomeres in aortic valves may participate in the development of AS, whereas at the same time the calcification process seems to induce a further decrease of TL in calcified areas. For a better understanding of the complex relation between telomere dynamics and the aortic valve calcification process, mechanistic studies are required.

## Data Availability Statement

The original contributions presented in the study are included in the article/Supplementary Material, further inquiries can be directed to the corresponding author/s.

## Ethics Statement

The studies involving human participants were reviewed and approved by the Stockholm Regional Ethical Committee. The patients/participants provided their written informed consent to participate in this study.

## Author Contributions

IS performed the experiments and wrote the first draft of the manuscript. AB carried out funding acquisition. IS, CL, and ST performed the data analysis. AF-C contributed to the collection of clinical samples. AB, MB, and ST designed the study, carried out the project administration, reviewed, and edited the manuscript. All authors read and approved the submitted version.

## Conflict of Interest

The authors declare that the research was conducted in the absence of any commercial or financial relationships that could be construed as a potential conflict of interest.

## Abbreviations

AS: aortic stenosis
ASCVD: atherosclerotic cardiovascular disease
CAVD: calcific aortic valve disease
LTL: leukocyte telomere length
TL: telomere length.
