# Supplementary material for: Telomere Length in Valve Tissue Is Shorter in Individuals With Aortic Stenosis and in Calcified Valve Areas
Source: Front Cell Dev Biol. 2021 Mar 11;9:618335. doi: 10.3389/fcell.2021.618335 (PMC7990782; doi:10.3389/fcell.2021.618335)
Supplement: Supplementary file 1 [file Data_Sheet_1.pdf]

Supplementary Material

Supplementary Figures

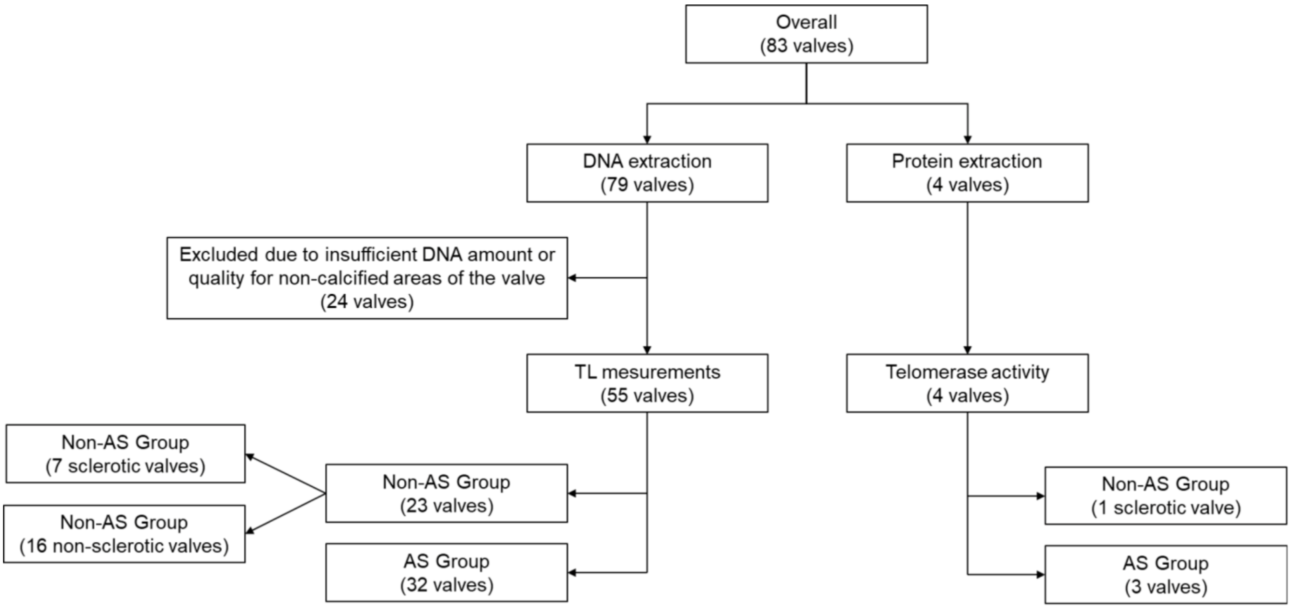

Supplementary Figure 1. Flow chart of experimental design. AS = aortic stenosis; TL = telomere length.

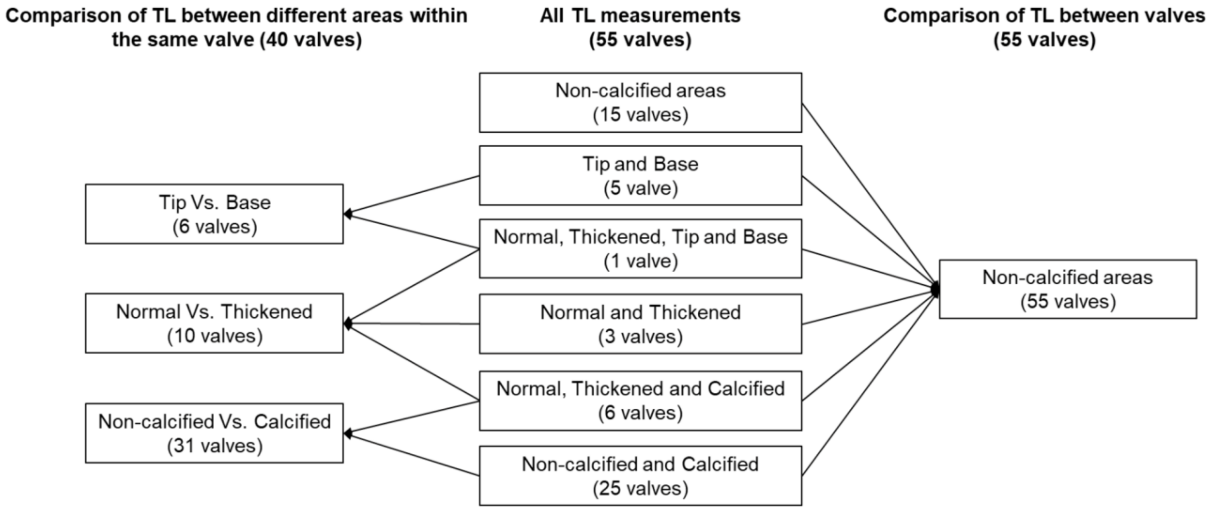

Supplementary Figure 2. Two approaches to analyse telomere length in different tissue categories. TL = telomere length.

A.

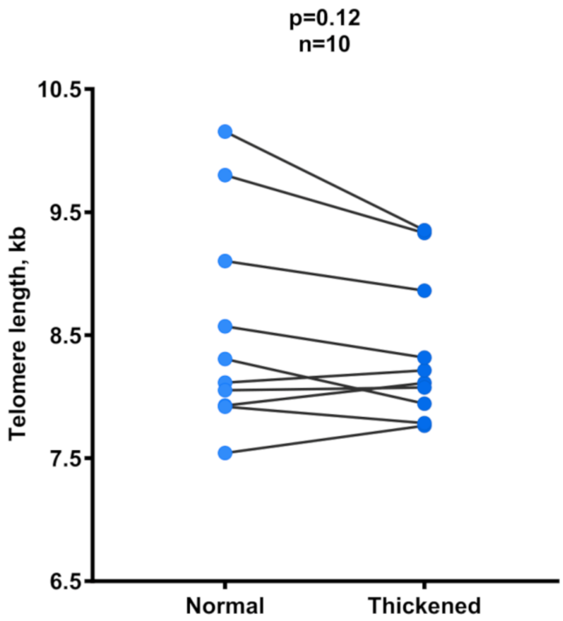

B.

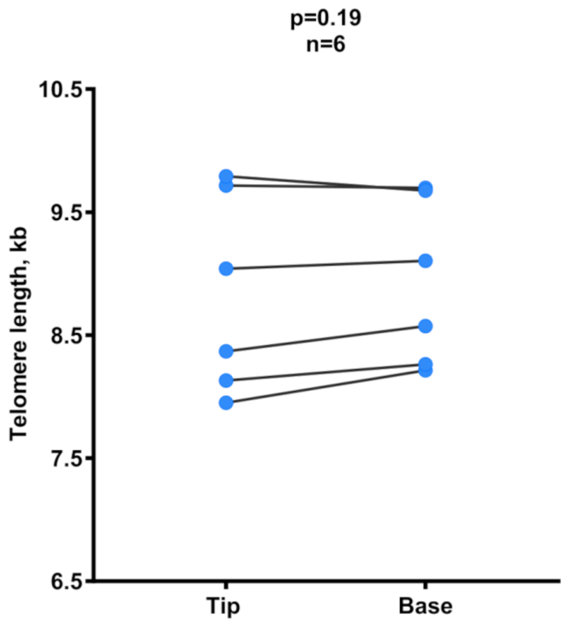

Supplementary Figure 3. Telomere length in different areas of non-calcified valves. **(A)** Telomere length in normal and thickened areas. **(B)** Telomere length in anatomical areas (tip and base) of valve. Matched circles indicate individual patients. Statistical analysis was performed using paired *t*-test. kb = kilobase.

A.

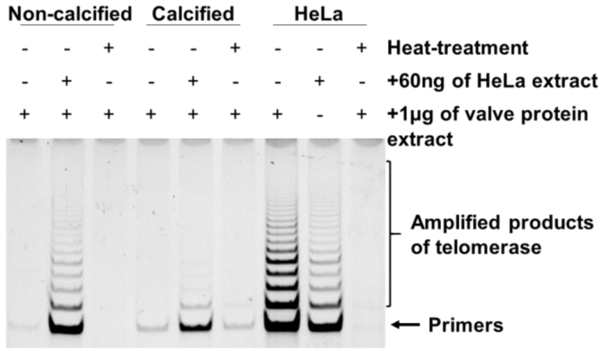

B.

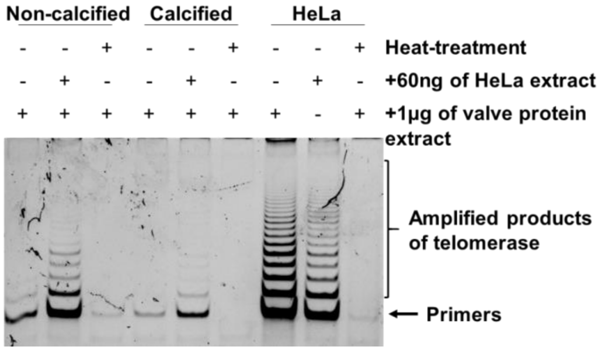

C.

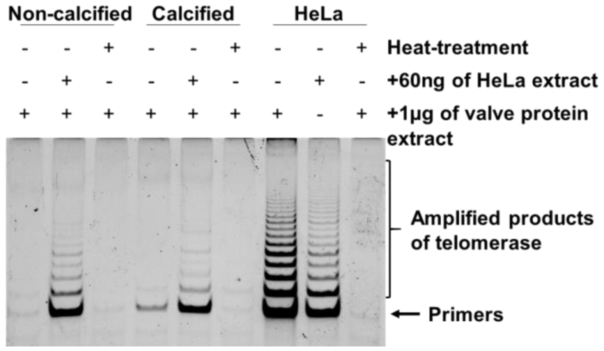

D.

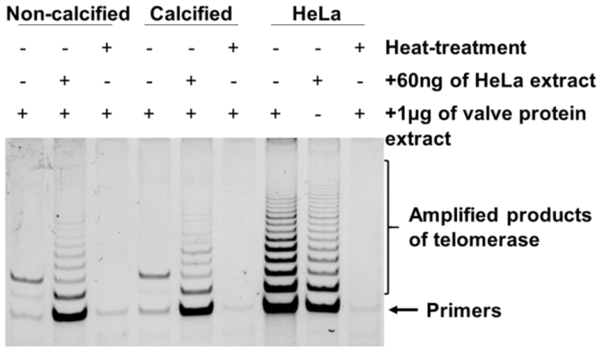

Supplementary Figure 4. Evaluation of telomerase activity by TRAP in non-calcified and calcified areas of aortic valves obtained from 4 patients. **(A)** 27 years old male with bicuspid valve and aortic sclerosis. **(B)** 70 years old female with tricuspid valve and aortic stenosis. **(C)** 64 years old male with bicuspid valve and aortic stenosis. **(D)** 57 years old female with bicuspid valve and aortic stenosis. Lines correspond to telomerase activity in 1µg of protein extract. Samples, which contain HeLa cell extract mixed with tissue extract, represent controls for false-negative results and heat-treated samples serve as controls for false-positive results. Extracts from HeLa cells were used as the positive control.

A. **B.**

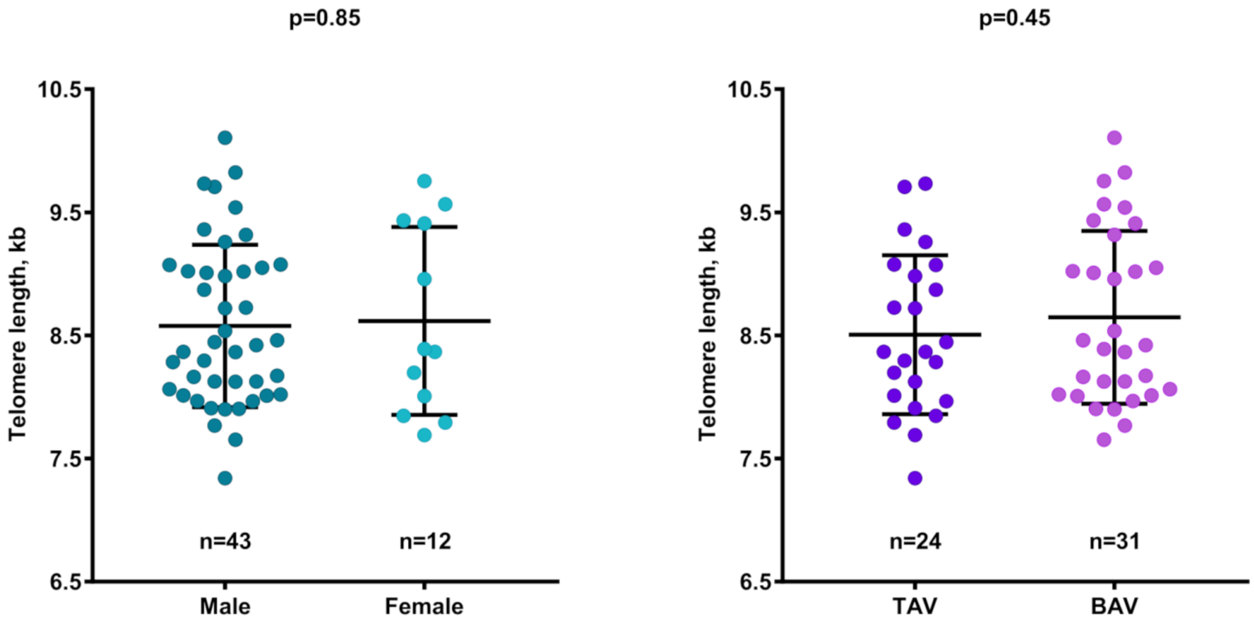

Supplementary Figure 5. Relation between telomere length in non-calcified valve tissues and sex and bicuspidy. **(A)** Telomere length in males and females. **(B)** Telomere length in triuspid and bicuspid aortic valves. Statistical analysis was performed using two-sample *t*-test. BAV = bicuspid aortic valve; kb = kilobase; TAV = tricuspid aortic valve.

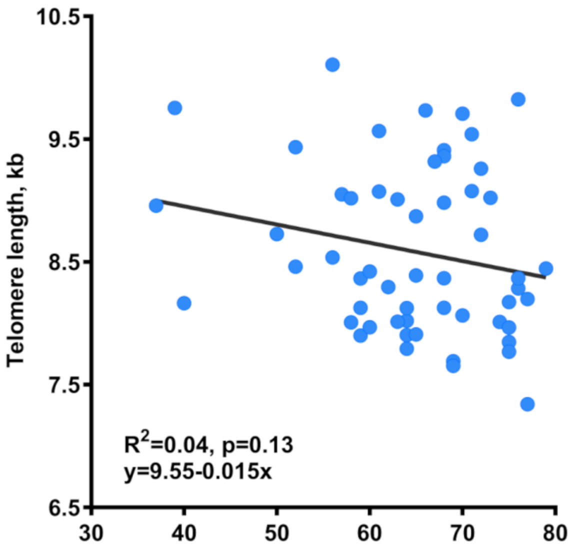

Supplementary Figure 6. Association of telomere length in non-calcified valve areas with age. R^2 and p-value are derived from Pearson correlation. kb = kilobase.
